# Screening for comorbid conditions in patients enrolled in the SODA registry: a 2-year observational analysis

**DOI:** 10.1007/s12020-018-1615-3

**Published:** 2018-05-16

**Authors:** Whitney W. Woodmansee, Murray B. Gordon, Mark E. Molitch, Adriana G. Ioachimescu, Don W. Carver, Beloo Mirakhur, David Cox, Roberto Salvatori

**Affiliations:** 1 Division of Endocrinology, Diabetes and Metabolism, University of Florida, 1600 SW Archer Road, Gainesville, FL 32610 USA; 2 Division of Endocrinology, Diabetes and Hypertension, Brigham and Women’s Hospital/Harvard Medical School, 221 Longwood Avenue, Boston, MA 02115 USA; 3 Allegheny Neuroendocrinology Center, Division of Endocrinology, Allegheny General Hospital, 420 E North Ave, Suite 205, Pittsburgh, PA 15212 USA; 4 Division of Endocrinology, Metabolism and Molecular Medicine, Northwestern University Feinberg School of Medicine, 645 N. Michigan Avenue, Suite 530, Chicago, IL 60611 USA; 5 Department of Medicine, Division of Endocrinology, Metabolism and Lipids, and Department of Neurosurgery, Emory University School of Medicine, 1365 B Clifton Rd, NE, B6209, Atlanta, GA 30322 USA; 6 Ipsen Biopharmaceuticals statistician consultant, 106 Allen Road, Basking Ridge, NJ 07920 USA; 7 Medical Affairs, Ipsen Biopharmaceuticals, Inc., 106 Allen Road, Basking Ridge, NJ 07920 USA; 8 Division of Endocrinology, Diabetes and Metabolism and Pituitary Center, Johns Hopkins University, 1830 East Monument Street #333, Baltimore, MD 21287 USA

**Keywords:** Acromegaly, Comorbidities, Extended-Release, Lanreotide Depot/Autogel, Observational Study, Registry

## Abstract

**Purpose:**

This 2-year analysis assessed frequency of comorbidities and comorbidity screening in the Somatuline^®^ (lanreotide, LAN) Depot for Acromegaly (SODA) registry.

**Methods:**

Patient data collected included pituitary hormone deficiencies, sleep studies, echocardiograms, gallbladder sonographies, colonoscopies, and glycated hemoglobin (HbA1c) levels. Insulin-like growth factor-1 (IGF-1) and growth hormone levels in patients with (DM) and without (non-DM) diabetes mellitus were analyzed.

**Results:**

There were 241 patients enrolled. Pituitary hormone deficiencies were reported more frequently at enrollment in male (56.9%) vs female patients (32.0%; *p* < 0.001). TSH deficiency was the most common endocrine deficiency (69.8%), followed by gonadotropin deficiency (62.3%). Screening tests reported at enrollment: sleep studies in 29.9% (79.2% had sleep apnea), echocardiogram in 46.1% (46.8% abnormal), gallbladder sonography in 18.7% (17.8% had gallstones), and colonoscopy in 48.1% (35.3% had polyps). Follow-up studies were reported less frequently at 1 and 2 years. HbA1c data were reported in 30.8% and 41.2% after 1 and 2 years. HbA1c levels were similar at 1 and 2 years of LAN therapy among DM and non-DM patients with available data. Fewer DM vs non-DM patients achieved IGF-1 below upper limit of normal at Month 24 (58.3% vs 80.6%; *p* = 0.033).

**Conclusions:**

Fewer than half of patients in SODA had screening results reported at enrollment for sleep apnea, cardiomyopathy, and colon polyps. Gallbladder imaging was reported in a minority of patients. Lower IGF-1 control rates were observed in DM vs non-DM patients at Month 24. These data suggest a need for better monitoring of comorbidities in US acromegaly patients.

## Introduction

Although acromegaly is a rare disease (United States [US] incidence was 11 cases per million person-years and prevalence was ~78 cases per million per year across 2008–2012) [1], the considerable burden of coexisting comorbidities and increased mortality represents a major medical problem [2–9]. Comorbidities significantly increase the odds of hospitalization and pharmacotherapy cost [10, 11]. Adequate management of acromegaly, including biochemical control and treatment of its major comorbidities, lowers the risk of mortality to the level of the general population [12–16], and may reduce healthcare utilization and cost associated with comorbidities [10, 11].

The Somatuline® (lanreotide) Depot for Acromegaly (SODA) registry is a post-marketing, multicenter, observational study of patients treated with lanreotide depot (LAN) in academic and private centers in the US (MS319, ClinicalTrials.gov Identifier: NCT00686348). Previous analyses of SODA have assessed the effectiveness, safety, and convenience of LAN in the treatment of acromegaly [17, 18]. Data from a 1-year analysis of the SODA registry showed that hormonal control was achieved independently of drug injection method, and patients found greater convenience with the use of self- or partner-injections [17]. A follow-up 2-year analysis showed that the majority of patients (54.8%) achieved both IGF-1 concentrations below upper limit of normal (<ULN) and GH levels ≤ 2.5 µg/L [18]. Disease control, defined as GH < 1.0 µg/L, was achieved in 61.4% of patients. Treatment-emergent adverse events (AEs) were reported in 54.4% of patients. The AE profile associated with LAN therapy was similar to the known safety profile of LAN and the somatostatin receptor ligands (SRLs).

Retrospective studies still show an ~70% increase in average standardized mortality rates in acromegaly patients compared with the general population [14, 15]. Therefore, addressing comorbidities is an important treatment goal. Various acromegaly treatment guidelines recommend evaluating all patients for associated comorbidities such as hypertension, diabetes mellitus (DM), cardiovascular disease, osteoarthritis, and sleep apnea, and include recommendations on managing comorbidities [2–7]. Screening for colon neoplasia, thyroid nodularity, and hypopituitarism is also recommended. For patients receiving SRLs, only those who develop signs and symptoms of gallstone disease should undergo abdominal ultrasound, and thus routine monitoring is not considered necessary [6]. Despite the availability of guidelines, the frequency with which clinicians screen and monitor comorbid conditions outside of controlled clinical trial settings is not well known. Using data from the SODA registry, the objective of this analysis was to summarize the frequency in which patients were assessed for acromegaly comorbidities in the US.

## Patients and methods

### Study design and patient population

Details of the SODA study design and patient population have been published previously [17, 18]. In brief, the patients were eligible for inclusion if they had a clinical diagnosis of acromegaly and either received LAN as their first medical therapy or were transitioned from other somatostatin analogs. The SODA study was conducted in accordance with the International Conference on Harmonisation Good Clinical Practice, current Food and Drug Administration regulations and guidelines, and local ethical and legal requirements. All data collection, transmission, and storage complied with the US Code of Federal Regulations and the Health Insurance Portability and Accountability Act. Signed informed consent was obtained from each patient at study inclusion.

Demographics, clinical characteristics, and comorbidities listed in the patient medical history were collected at enrollment. Patients could be enrolled before or after starting LAN. Pituitary hormonal deficiencies were reported by the investigator using a checkbox format for ACTH, ADH, TSH, and gonadotropin deficiencies. Menstrual cycle and menopausal status were not recorded. Hypopituitarism was defined as a deficiency in ≥1 of the 4 recorded pituitary hormonal deficiencies. Data from optional speciality tests, including sleep study, echocardiogram, gallbladder sonography, and colonoscopy were recorded as obtained during clinic visits at the discretion of the treating physician. Biochemical control [18] was assessed using serum IGF-1 and random serum GH levels (both evaluated mostly at local laboratories). Since serum GH levels at enrollment were not uniformly available, GH control was only assessed at Month 12 (M12) and M24 and only for patients not on pegvisomant. Control of glucose homeostasis was assessed using glycated hemoglobin (HbA1c) levels, when available, at thresholds of <5.7% (in reference range), 5.7%–6.4% (pre-DM), and ≥6.5% (DM). Since HbA1c levels at enrollment were not uniformly available, HbA1c control was assessed at M12 and M24 only. A patient was defined as having diabetes based on medical history, anti-diabetic medication use, and/or HbA1c level ≥6.5%.

### Statistical analysis

Data for this analysis reflect an interim cut-off as of 29 September 2014. Continuous data were analyzed using descriptive statistics (mean, median, and standard deviations). Student’s *t*-tests were performed for exploratory purposes. Categorical data were described by frequencies and by chi-square or Fisher’s exact test probabilities, also performed for exploratory purposes. Probability tests of categorical data are considered to be chi-square unless noted otherwise. A two-sided *p* < 0.05 was considered significant. *P*-values were not adjusted for multiple comparisons. The study population included all patients who received a dose of study drug. This was an observational study, and thus the frequency of study visits and assessments occurred at the discretion of the clinician, which resulted in different sets of patients across visits (unpaired) and reflected the availability of reported data.

## Results

As of 29 September 2014, 241 patients were enrolled and included in the 2-year dataset [18]. Sixty-one patients were <40 years of age, 124 were 40–60 years of age, and 56 were >60 years of age. Acromegaly was caused by pituitary adenoma in 97% of patients (233/241); 8 patients had other causes, including tumor not identified (*n* = 2), tumor secreting GH-releasing hormone (*n* = 1), McCune-Albright syndrome (*n* = 1), and cause not specified (*n* = 4). The median time since acromegaly diagnosis was 4.3 years (quartile [Q]1 = 1.71 years, Q3 = 9.25). Additional demographic and clinical characteristics of the 2-year SODA population can be found in the report by Salvatori et al [18].

Patient comorbidities at enrollment are summarized in Table 1. Almost half (48.6%) of patients at enrollment had cardiovascular comorbidities, with hypertension being most prevalent (45.2%). DM was present in 25.3% of patients at enrollment. Despite the slight predominance of female patients with acromegaly in SODA, one or more pituitary hormone deficiencies were reported more frequently at enrollment in male (66/116, 56.9%) vs female patients (40/125, 32.0%; *p* < 0.001) (Table 1). Details regarding specific hormone deficiencies were available in 106 patients with hypopituitarism; most had single hormone deficiencies (61/106, 57.5%), while 25.5% (27/106), 13.2% (14/106), and 3.8% (4/106) had 2, 3, and 4 hormone deficiencies, respectively. TSH deficiency was the most common pituitary hormone dysfunction (69.8%, 74/106). Gonadotropin deficiency was reported in 66 of the patients with hypopituitarism (62.3%; 66/106): 57 males (86.4%; 57/66) and 9 (13.6%; 9/66) females (*p* < 0.001). Of the 125 females in the study, only 9 (7.2%) were reported to have gonadotropin deficiency and of these, 5 were treated with estrogen. The rates of reported gonadotropin deficiency were similar between women above age 50 (7%; 5/71) and below age 50 (7.4%; 4/54). Adrenocorticotropic hormone (ACTH) deficiency occurred in 28 patients. Of the 241, 5 (2.1%) patients had diabetes insipidus, and all 5 had previous pituitary surgery.

**Table 1:** Patient characteristics and comorbidities in acromegaly patients in the SODA study at enrollment

Characteristic/comorbidity^a^	Male (*n* = 116)	Female (*n* = 125)	All patients (*N* = 241)	*p*-value
Age, years; mean ± SD (range)	47.4 ± 13.5 (17–77)	52.1 ± 15.0 (13–86)	49.8 ± 14.4 (13–86)	0.011
**Cardiovascular** ^b^	53 (45.7)	64 (51.2)	117 (48.6)	0.392
Arterial hypertension	49 (42.2)	60 (48.0)	109 (45.2)	
Coronary artery disease	7 (6.0)	7 (5.6)	14 (5.8)	
Peripheral edema	4 (3.5)	4 (3.2)	8 (3.3)	
Congestive heart failure	3 (2.6)	2 (1.6)	5 (2.1)	
Angina pectoris	3 (2.6)	1 (0.8)	4 (1.7)	
Arrhythmias	3 (2.6)	0	3 (1.2)	
Cardiomyopathy	3 (2.6)	1 (0.8)	4 (1.7)	
Myocardial infarction	1 (0.9)	1 (0.8)	2 (0.9)	
**Hypopituitarism** ^b^	66 (56.9)	40 (32.0)	106 (44.0)	<0.001
TSH deficiency	37 (31.9)	37 (29.6)	74 (30.7)	
Gonadotropin deficiency	57 (49.1)	9 (7.2)	66 (27.4)	
ACTH deficiency	18 (15.5)	10 (8.0)	28 (11.6)	
ADH deficiency	3 (2.6)	2 (1.6)	5 (2.1)	
**Arthralgia/arthritis**	42 (36.2)	48 (38.4)	90 (37.3)	0.725
**Lipid abnormalities**	37 (31.9)	43 (34.4)	80 (33.2)	0.680
**Diabetes mellitus**	23 (19.8)	38 (30.4)	61 (25.3)	0.059
**Carpal tunnel syndrome**	20 (17.2)	18 (14.4)	38 (15.8)	0.545
**Malignancies** ^b^	6 (5.2)	20 (16.0)	26 (10.8)	0.007
Thyroid carcinoma	1 (0.9)	5 (4.0)	5 (2.1)	
Breast	0	4 (3.2)	4 (1.7)	
Skin (melanoma, 2; basal cell carcinoma, 1; skin malignancy not specified, 1)	2 (1.7)	2 (1.6)	4 (1.7)	
Brain (brain cell glioma, 1; glioblastoma, 1; meningioma, 1)	0	3 (2.4)	3 (1.2)	
Blood (Burkitt lymphoma, 1; lymphoma, 1)	1 (0.9)	1 (0.8)	2 (0.8)	
Prostate	2 (1.7)	0	2 (0.8)	
Mandibular	1 (0.9)	0	1 (0.4)	
Lung	1 (0.9)	0	1 (0.4)	
Cervix uteri	0	1 (0.8)	1 (0.4)	
**Psychosocial** ^b^	6 (5.2)	15 (12.0)	21 (8.7)	0.060
Depression	5 (4.3)	10 (8.0)	15 (6.2)	
Anxiety	1 (0.9)	8 (6.4)	9 (3.7)	
**Cerebrovascular (stroke)**	4 (3.5)	5 (4.0)	9 (3.7)	1.000^c^
**Osteopenia/osteoporosis**	4 (3.5)	5 (4.0)	9 (3.7)	1.000^c^
**Kidney stones**	3 (2.6)	1 (0.8)	4 (1.7)	0.354^c^
**Pancreatitis**	1 (0.9)	2 (1.6)	3 (1.2)	1.000^c^

Data are given as *n* (%) unless otherwise noted; *p*-values for comparisons between male and female patients are determined by chi-square unless otherwise noted

^a^Includes comorbidities assessed at enrollment and those listed in patient medical history

^b^Not mutually exclusive; number of patients with individual comorbidities in a category (bold) may be larger than the number in the overall category. TSH deficiency does not include primary thyroid disease.

^c^*p*-value determined by Fisher’s exact test

*ACTH* adrenocorticotropic hormone, *ADH* antidiuretic hormone, *TSH* thyroid-stimulating hormone

**Fig. 1:**
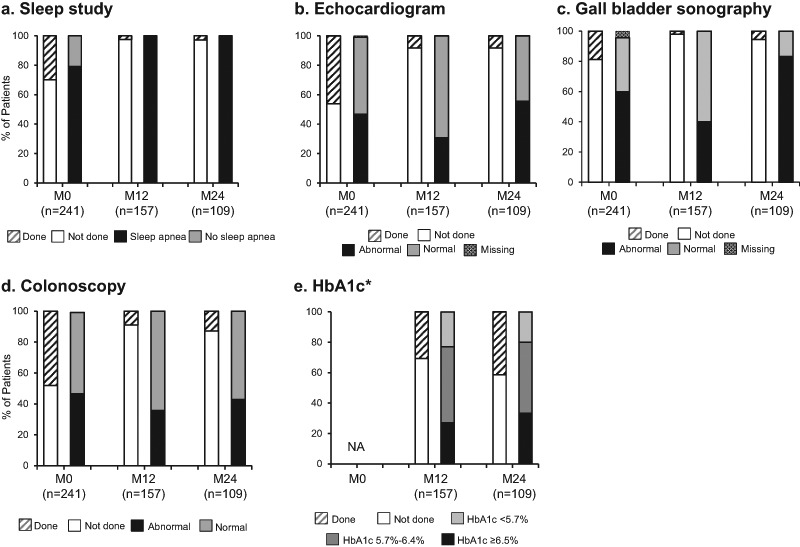
Optional speciality tests (except HbA1c) at enrollment and follow-up assessments in the SODA study. Data in the first bar of each time category include all available patients, and data in the second bar of each category include only those patients who had a test. HbA1c, glycated hemoglobin; M0, Enrollment; M12, Month 12; M24, Month 24; NA, not available. *Total group, includes patients with and without diabetes

Data from the optional speciality tests conducted at enrollment and during SODA and assessments of glucose homeostasis during SODA are shown in Fig. 1.

### Screening for sleep apnea: sleep study

At the enrollment visit, 29.9% of patients (72/241) had a sleep study recorded, with the majority of these showing sleep apnea (Fig. 1a). At M12 and M24, 2.5% (4/157) and 2.8% (3/109) of patients had a sleep study (new screenings each, not follow-up), respectively, with 100% showing sleep apnea.

### Screening for cardiac abnormalities: echocardiogram

At enrollment, 46.1% of patients (111/241) had an echocardiogram; nearly one-half of these (46.8%, 52/111) were abnormal (Fig. 1b). Abnormalities included ventricular hypertrophy/enlargement in 19.8% (22/111), valve disorders in 10.8% (12/111), diastolic/systolic dysfunction in 6.3% (7/111), pulmonary hypertension in 2.7% (3/111), and other/not specified in 7.2% (8/111). At M12 and M24, echocardiogram was performed in 8.3% of patients (13/157 and 9/109, respectively), with 4 and 5 patients having abnormal results, respectively. Two patients had repeat echocardiograms at M12 and M24. Of interest, a total of 39 patients had both an echocardiogram and a polysomnogram at baseline. Of the patients with both studies (*n* = 39), 19 had an abnormal echocardiogram at baseline. For these patients with an abnormal echocardiogram, 94.7% (18/19) had a sleep study showing sleep apnea compared to 11.1% (1/19) without sleep apnea (*p* = 0.02, Fisher’s exact test).

### Screening for gallbladder stones: gallbladder sonography

Of the 18.7% of patients (45/241) who had gallbladder sonography results recorded at enrollment, 60% (27/45) had abnormal findings (Fig. 1c). Abnormalities included gallstones in 17.8% (8/45) (small in 7, large in 1), sludge in 24.4% (11/45), and other abnormalities in 33.3% (15/45) of patients. At M12, 3.2% of patients (5/157) had gallbladder sonography, with 2 patients having abnormal findings (other abnormalities). At M24, 5.5% of patients (6/109) had gallbladder sonography, with 5 having abnormalities, including 3 patients each with gallstones (small in 2, large in 1) and other abnormalities.

### Screening for colon polyps: colonoscopy

Approximately half (48.1%, 116/241) of patients had colonoscopy results recorded at enrollment, and the 65 patients with colonoscopy dates recorded had one within ~3 years of enrollment. Abnormalities were found in 46.6% (54/116) (Fig. 1d), including polyps in 35.3% (41/116) of patients and other abnormalities in 12.1% (14/116) of patients. At M12, 8.9% of patients (14/157) had colonoscopy, with 5 patients showing abnormalities, including 3 patients with polyps and 2 patients with other abnormalities. At M24, colonoscopy was performed in 12.8% (14/109) of patients, with 6 showing abnormalities, including polyps in 6 patients and other abnormalities in 1 patient. No cancers were indicated in any of the abnormal findings.

### Glucose homeostasis and biochemical control: diabetes and HbA1c

**Fig. 2:**
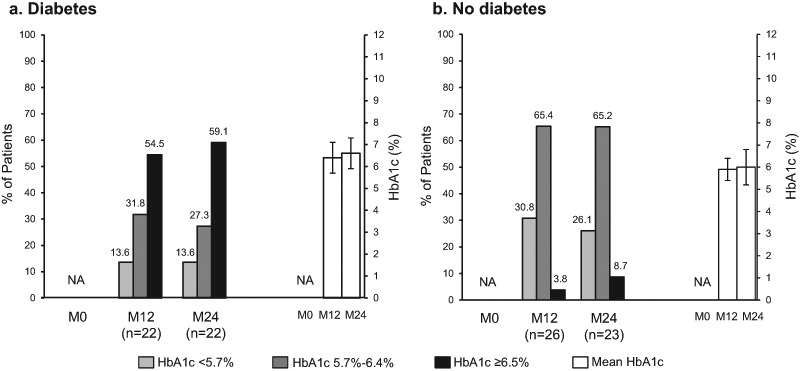
Proportion of acromegaly patients with (**a**) and without (**b**) diabetes in each HbA1c threshold, and mean HbA1c levels (mean ± standard deviation) in the SODA study. HbA1c, glycated hemoglobin; M0, Enrollment; M12, Month 12; M24, Month 24; NA, not available

**Fig. 3:**
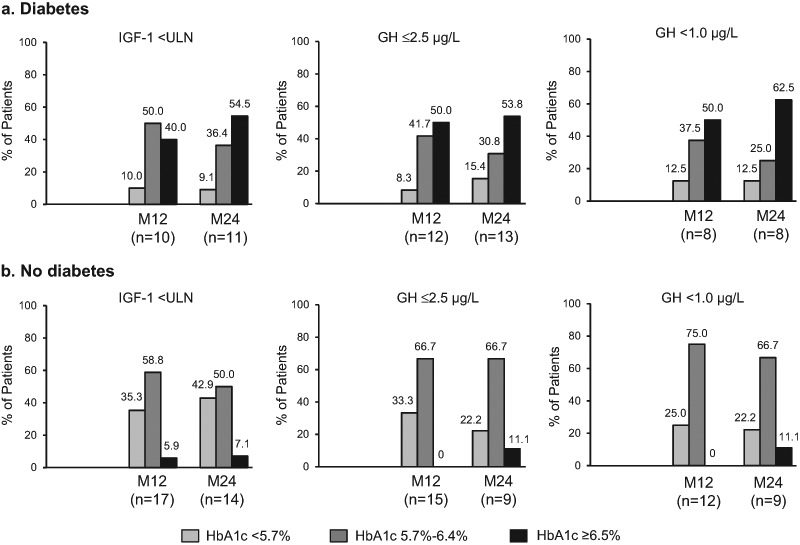
Proportion of biochemically controlled acromegaly patients with (**a**) and without (**b**) diabetes at each HbA1c threshold in the SODA study. GH control analyses excluded patients who were taking pegvisomant. GH, growth hormone; HbA1c, glycated hemoglobin; IGF-1 insulin-like growth factor-1, ULN, upper normal level for age and gender; M12, Month 12; M24, Month 24

HbA1c was measured in 48/157 patients (30.6%) at M12 and 45/109 (41.3%) at M24 (Fig. 1e). DM patients were analyzed for HbA1c in 51.2% (22/43) at M12, and 71.0% (22/31) at M24, and for non-DM patients in 22.8% (26/114) at M12, and 29.5% (23/78) at M24. Mean HbA1c levels among all patients with available data were similar at M12 and M24 within each DM and non-DM group. Within each DM and non-DM group, the proportion of patients with reported HbA1c <5.7%, 5.7%–6.4%, and ≥6.5% at M12 and M24 were also similar (Fig. 2). A sensitivity analysis was conducted to ascertain the impact of pegvisomant on glucose control in the patients treated with lanreotide. In diabetic patients (*n* = 22), none were receiving pegvisomant, so no comparative analysis was possible. In non-diabetic patients (*n* = 23), only 3 patients were receiving concomitant pegvisomant with 66.7% (*n* = 2/3) reporting a HbA1c between 5.7%–6.4%, compared to 65.2% (*n* = 15/23) of those not receiving pegvisomant reporting a HbA1c in the same range. Also within each DM and non-DM group, the proportions of biochemically controlled patients reported within each HbA1c level at M12 and M24 were similar (Fig. 3).

**Fig. 4:**
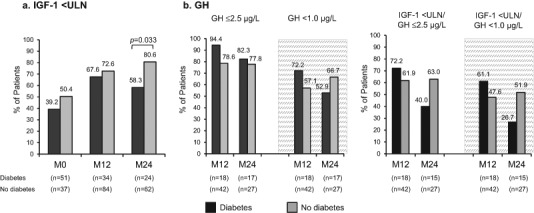
Biochemical control in acromegaly patients with and without diabetes in the SODA study. **a** IGF-1 <ULN; **b** GH ≤2.5 µg/L, and GH <1.0 µg/L (gray area); **c** Both IGF-1 <ULN/GH ≤2.5 µg/L and IGF-1 < ULN/GH <1.0 µg/L. GH control analyses excluded patients who were taking pegvisomant. GH, growth hormone; IGF-1, insulin-like growth factor-1; ULN, upper normal level for age and gender; M0, Enrollment; M12, Month 12; M24, Month 24. Panel 4a appeared as panel 1f and panel 4b appeared as panel 3f/4f in Salvatori R, et al (2017) [18]. Used with permission

Biochemical control was reported among the majority of all patients with available data in the DM and non-DM groups at M12 and M24 (Fig. 4). Though the proportion of patients achieving IGF-1 control did not differ between DM and non-DM at enrollment and M12, the study revealed lower rates of IGF-1 <ULN in the DM vs non-DM group at M24 (*p* = 0.033).

There were no differences between the DM and non-DM groups in proportion of patients achieving GH ≤2.5 µg/L at M12 and M24, and GH <1.0 µg/L at M12 and M24 (Fig. 4). Similarly, no differences were revealed in biochemical control of both IGF-1 and GH levels in the DM vs non-DM group.

**Fig. 5:**
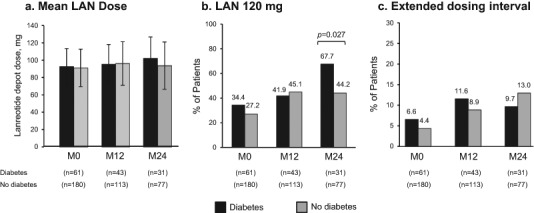
LAN dose and regimen in acromegaly patients with and without diabetes in the SODA registry: **a** Mean 28-day dose equivalent (M ± SD); **b** 120 mg; **c** Extended dosing interval. M0, Enrollment; M12, Month 12; M24, Month 24; LAN, lanreotide

Mean LAN 28-day dose equivalent use did not differ between the DM and non-DM groups at M12 and M24 (Fig. 5). A greater proportion of DM vs non-DM patients received LAN 120 mg at M24 (*p* = 0.027). Reported LAN extended dosing interval (EDI) use did not differ between the DM and non-DM groups at M12 and M24. There was no significant difference in IGF-1 control between LAN mono- and combination therapy except in non-DM patients at M12, where those receiving LAN monotherapy achieved significantly better IGF-1 control vs combination therapy (44/54, 81.5% vs 17/30, 56.7%; *p* = 0.015).

### Patient deaths

AEs recorded during the 2-year study period have been reported [18]. Four patients (2 male, 2 female) died during the 2-year observation (*n* = 1 each, congestive heart failure, heart and respiratory failure, cardiac arrest, and not otherwise specified); all deaths were considered by the investigator to be unrelated to treatment. Comorbidities among these 4 patients included diabetes in 1, hypertension in 4, and hyperlipidemia in 2. None of the patients had ACTH deficiency.

## Discussion

In keeping with guidelines and consensus statements on the diagnosis and treatment of acromegaly-associated comorbidities [2, 6, 7], this report examined the frequency of monitoring for comorbidities and described the findings in a cohort of patients with acromegaly from the SODA registry. The frequency of optional testing and comorbidity screening recorded at enrollment, 1, or 2 years was low overall, potentially reflecting the ongoing challenges and geographic differences in managing this population of patients. Approximately 50% or fewer of patients had results from sleep studies, echocardiograms, gallbladder sonographies, or colonoscopies during study enrollment, and overall follow-up was infrequently reported during the 2-year observation period despite abnormal test results in some patients at enrollment. The reasons for the low frequency of testing for comorbidities are not exactly clear, but may involve differences in assessment practices for patients with a history of acromegaly vs patients at the time of initial diagnosis. Also, practitioners may not be performing certain follow-up assessments if a patient with a history of acromegaly does not also have a history of the comorbid condition. Furthermore, if a patient achieves and sustains target biochemical treatment goals, they may not be perceived of as being at an increased risk for some of the associated comorbid conditions, even though evidence has shown that some potentially severe comorbidities, including arthropathy and sleep apnea, may persist even after long-term biochemical control of acromegaly [19, 20]. Approximately two-thirds of the patients enrolled in the SODA study were recruited from sites in academic medical centers and one-third were from community private practices [17]. However, whether the number of recruited patients per study site was proportional to the volume of acromegalic patients cared for at a particular location is not known. Therefore, no conclusions can be made regarding the frequency of testing for comorbidities and the volume of acromegalic patients seen in a particular center. Limitations attributed to the observational study design may have also contributed to the low frequencies of testing reported. Specifically, patients may have had a particular study performed but the result was either unknown or not recorded in the SODA database by the investigator. In addition, the timeframe of the SODA study should be considered, as the study used a data cut-off of 2014, which was prior to the publication of the most recent acromegaly management guidelines. It should be noted, however, that the incidence of comorbidities was similar and in line with that seen in other observational studies of patients with acromegaly [21, 22].

Cardiovascular comorbidities, including hypertension (predominantly diastolic), left ventricular hypertrophy (LVH), diastolic and systolic dysfunction, arrhythmias, myocardial infarction, enlarged great vessel diameters, and valve diseases are highly prevalent in acromegaly and are considered a major cause of mortality [12, 14–16, 23–27]. Among them, hypertension [16] and cardiomyopathy [24] are considered the main cardiac risk factors that directly impact mortality. Three of 4 deaths in SODA resulted from cardiovascular complications, and all 4 patients had hypertension. Hypertension in SODA was documented in almost half (45.2%) of patients at enrollment, consistent with 47.5% in the US acromegaly registry reported by the Pituitary Center at Cedars-Sinai Medical Center (CSMC-PC) [28] but higher than rates documented in other studies, including registry studies (rates ranging from 22% to 41.3%) [9, 14, 21–24, 29, 30]

The prevalence of different features of cardiomyopathy is 3.3–14.2 times higher in the acromegalic than non-acromegalic population, with the disease duration as its major determinant [24, 31]. Guidelines recommend routine echocardiogram and electrocardiogram at diagnosis and annually during follow-up, especially in patients who have evidence of ventricular hypertrophy by electrocardiography or who are symptomatic, particularly if older [2, 6]. In SODA, only 3 (1.2%) patients had documented cardiomyopathy at enrollment based on patient medical histories. Taking into account 4.3 years as the median time since acromegaly diagnosis, we can assume that cardiomyopathy was under-assessed in this cohort of patients. Indeed, just under half of the patients had an echocardiogram at enrollment, and very few patients had one at follow-up visits (<10%), of whom nearly half had abnormal findings. These data suggest that, in real-world practice, guideline recommendations are not always followed. However, according to the Endocrine Society 2014 Acromegaly Guidelines, the role of pretreatment echocardiogram has not been defined; rather, thorough cardiac evaluation may be indicated by suggestive clinical findings, particularly in perioperative patients [6]. Also, according to a cardiac MRI study, patients with active acromegaly might have a lower (5%) prevalence of LVH than previously reported using echocardiogram [32].

Sleep apnea, primarily obstructive, is a frequent comorbidity in acromegaly due to soft tissue thickening and edema of the tongue, pharynx, and upper airways, with a reported prevalence of 25%–60% [5] and ~69% [6]. Although biochemical control of acromegaly usually improves sleep apnea, it has been shown to persist in ~40% of biochemically controlled patients [6, 33]. Guidelines recommend an annual Epworth Sleepiness Scale or sleep study during follow-up of acromegaly [2–4, 6]. Less than one-third of patients had completed a sleep study at SODA enrollment, and very few (<3%) had entered a sleep study at follow-up visits. At enrollment, the majority of tested patients had sleep apnea (79.2%), consistent with other reports [33–35], although the CSMC-PC and ACROSTUDY reported significantly lower (22.5% and 17%, respectively) prevalence of sleep apnea [22, 28]. However, these numbers are not strictly comparable, since symptomatic patients were more likely to get sleep studies, and thus the true prevalence may not be reflected. Also, the SODA database did not distinguish between obstructive or central sleep apnea. Nonetheless, given that sleep apnea is independently associated with hypertension and cardiovascular disease [33, 34] and proposed to account for up to 25% of the excess mortality in untreated acromegaly [33], there may be potential for improved outcomes in acromegaly patients if sleep apnea screening is increased.

The frequent prevalence of DM in SODA patients (25.3%), along with older-age, higher-BMI, and higher coexistence of hypertension and statin use in DM vs non-DM patients noted in the previous 2-year SODA analysis [18], were findings similar to those reported in several other studies [6, 9, 22] and registries [21, 28, 29, 36]. Hyperglycemia at diagnosis and ongoing SRL treatment are independent predictive factors of persistent or new glucose abnormalities during follow-up [37]. In addition, patients with comorbid DM are reported to have a lower survival rate than patients without DM [38]. Therefore, all patients with acromegaly should be tested for glucose intolerance and DM, appropriately treated, and followed up as indicated for these conditions. Guidelines recommend an oral glucose tolerance test at diagnosis, fasting blood glucose every 6 months (particularly in uncontrolled disease and during SRL therapy), and HbA1c every 6 months if diabetes is present [2–4, 6]. In SODA, less than half of patients had HbA1c results reported after 1 and 2 years of LAN therapy.

Lowering GH levels improves glycemic control and increases insulin sensitivity in acromegaly; however, SRL therapy may exert variable effects on glucose metabolism, with worsening due to inhibition of insulin secretion and improvement due to improved insulin sensitivity with acromegaly control [39, 40]. Although the number of patients with reported HbA1c levels in this SODA analysis were small, similar proportions of patients were reported in each HbA1c level at M12 and M24 within each DM and non-DM subgroup. These findings support other reports of the relatively minor impact of SRLs on glucose homeostasis [39], in particular LAN [41, 42], which is less detrimental to diabetes than the next-generation SRL pasireotide [43].

Reported biochemical control rates were similar among patients at M12 and M24, independent of glucose homeostasis levels. No significant differences between DM and non-DM groups were observed in patients who achieved both ≤2.5 µg/L and <1.0 µg/L GH control after 1 and 2 years of treatment. Conversely, the data revealed higher rates of IGF-1 <ULN in non-DM vs DM patients after 2 years of treatment. The similar mean LAN 28-day dose equivalent and LAN EDI use, and higher use of the 120-mg LAN dose among DM vs non-DM patients, suggests that inadequate LAN dosing would not account for the higher IGF-1 levels in the DM group at M24. One likely explanation is the hyperinsulinism in the DM group, which enhanced synthesis of IGF-1 through upregulation of hepatic GH receptors [17, 44, 45]. The disconnect between the similar GH levels in DM vs non-DM patients and the difference in IGF-1 may also be explained by the effect of obesity [45] and hyperinsulinism both increasing hepatic GH sensitivity [46] and increasing free fatty acids, which suppress GH release [47]. These findings are in agreement with another recent analysis of SODA data, which indicated that more obese vs non-obese patients achieved GH <1.0 µg/L after both 1 and 2 years of LAN treatment [18]. Notably, this trend of GH control was opposite to IGF-1 control. These data are in line with those of Matta et al [48], who report metabolic syndrome markers such as higher fasting blood glucose and systolic blood pressure, in treated patients with acromegaly with a high IGF-1 and GH <1 µg/L.

In some studies, colon polyps and/or cancer have been reported to occur more frequently in acromegaly than in the general population, although data from other studies have not supported this association [49, 50]. There is general agreement, however, that early colonoscopy screening and regular surveillance is justified [38, 49, 51–54]. The Screening Guidelines for Colorectal Cancer and Polyps in Acromegaly [52] recommend regular colonoscopic screening, starting at 40 years of age, with frequency of repeat colonoscopy depending on findings at original screening and acromegaly activity. Almost half of SODA patients had undergone a colonoscopy by enrollment, many within 3 years, and 35.3% had polyps. These findings are higher than in other studies [9, 10, 55] and in the Belgian (27.2%) [21] and CSMC-PC (20.0%, polyps or colon cancer) [28] registries. Follow-up colonoscopies were recorded in fewer numbers of SODA patients.

Although the impact of acromegaly and its control on neoplasia risk and mortality are controversial [6, 8, 27], the presence of cancer and the last IGF-1 level are considered significant mortality predictors in acromegaly [9, 12, 38]. Malignancies of various systems were reported in 10.8% of SODA patients at enrollment, which is consistent with 10.5% in the Belgian registry [21]. Thyroid cancer is a commonly reported cancer in acromegaly[6, 9], followed by breast, lung, ovarian, and lymphoma [9]. Consistent with these reports, thyroid, skin, and breast were the most common cancer types in the SODA cohort, followed by brain, blood (lymphoma), and prostate.

Hypopituitarism in acromegaly may develop due to tumor compression or as a result of surgical or radiation treatment. Acromegaly guidelines recommend assessing for hypopituitarism and adequate replacement of adrenal, gonadal, and thyroid insufficiency; patients who receive radiotherapy need lifelong monitoring of pituitary function [2, 4, 6]. In SODA, 44.0% of enrolled patients presented with pituitary hormone deficiencies, which is higher than the rates reported in several European registries [21, 30, 56]. Similar to the German registry [56], pituitary insufficiency was more commonly reported in males compared to females. The prevalences of TSH and gonadotropin deficiencies were higher in SODA than in the German registry[56], CSMC-PC registry [28], and ACROSTUDY [22]. Interestingly, as in the German registry [56], gonadotropin deficiency in SODA was more commonly reported in males (49.1%) vs females (7.2%). The cause for this apparent gender difference is unknown but it is important to consider in registry studies that it may be due to ascertainment bias or potentially less rigorous documentation of gonadal status in women. This gender difference does not appear to be related to tumor size, surgery, or radiation exposure as there were no gender differences noted for these variables in SODA [18]. ACTH deficiency in SODA (11.6%) was similar to the German registry (11.8%) [56], but lower than in the CSMC-PC registry (14.9%) [28] and ACROSTUDY (15%) [22]. ACTH deficiency can lead to adrenal crisis during acute illness and may cause adverse metabolic effects due to chronic supra-physiological glucocorticoid replacement, which are associated with increased mortality in patients with acromegaly [2, 27, 56, 57]. None of the 4 patients who died in SODA had ACTH deficiency.

Gallbladder stones were present in 17.8% of SODA patients with a gallbladder sonography performed at enrollment, which falls between the rates reported in the ACROSTUDY (7%) [22] and Belgian registry (23.4%) [21]. Of note, gallbladder sonography was performed in only 18.7% of SODA patients. The Endocrine Society guideline does not suggest routine abdominal ultrasound to monitor for gallstone disease in a patient receiving an SRL [6]; however, ultrasound should be performed if a patient has signs and symptoms of gallstone disease. In SODA, the frequency of gallbladder ultrasound was relatively low throughout the observation period, which could be in part because the study protocol excluded patients with symptomatic, untreated biliary lithiasis. Moreover, a retrospective case cohort study of 31 consecutive newly diagnosed patients with acromegaly revealed a significantly increased prevalence of gallbladder polyps compared with a control group [58].

Given that SODA is an observational study, heterogeneity of data collection across centers is an inherent limitation, in addition to the fact that patients in SODA were enrolled at different stages of disease and treatment. Follow-up in observational studies is generally not as active or as standardized as in randomized trials; therefore, ascertainment of outcomes may be incomplete or inaccurate. The optional studies recorded in the database may not represent a completely accurate account of all the studies actually performed due to the possibility of under-reporting. Although registries are typically more generalizable to ‘real world’ practice because of their observational design, entry into a registry may not be as strictly monitored compared with randomized trials. This may weaken the generalizability of findings obtained from analysis of registry data. The subgroup comparisons were also limited by small sample sizes in some instances.

In conclusion, this analysis of acromegaly patients from the observational SODA registry found less frequent real-life monitoring of comorbid conditions than recommended by recent treatment guidelines. Additional prospective analysis will be needed to further identify and address potential barriers to managing acromegaly-associated comorbidities and the impact of screening on survival.
